# Ebolavirus Evolution: Past and Present

**DOI:** 10.1371/journal.ppat.1005221

**Published:** 2015-11-12

**Authors:** Marc-Antoine de La Vega, Derek Stein, Gary P Kobinger

**Affiliations:** 1 Special Pathogens Program, National Microbiology Laboratory, Public Health Agency of Canada, Winnipeg, Manitoba, Canada; 2 Department of Immunology, University of Manitoba, Winnipeg, Manitoba, Canada; 3 Department of Medical Microbiology, University of Manitoba, Winnipeg, Manitoba, Canada; 4 Department of Pathology and Laboratory Medicine, University of Pennsylvania School of Medicine, Philadelphia, Pennsylvania, United States of America; University of Alberta, CANADA

## Abstract

The past year has marked the most devastating Ebola outbreak the world has ever witnessed, with over 28,000 cases and over 11,000 deaths. Ebola virus (EBOV) has now been around for almost 50 years. In this review, we discuss past and present outbreaks of EBOV and how those variants evolved over time. We explore and discuss selective pressures that drive the evolution of different Ebola variants, and how they may modify the efficacy of therapeutic treatments and vaccines currently being developed. Finally, given the unprecedented size and spread of the outbreak, as well as the extended period of replication in human hosts, specific attention is given to the 2014–2015 West African outbreak variant (Makona).

## Introduction

Filoviruses are negative-sense, single-stranded RNA viruses. Depending on the virus and the variant involved, case fatality rates in outbreak settings can vary between 25% and 90%. Almost 50 years ago, the first member of the *Filoviridae* family, Marburg virus (MARV), was identified following two simultaneous outbreaks in Marburg, Germany, and Frankfurt, Germany, as well as in Belgrade, Serbia (former Yugoslavia) [1]. It was not until 1976 that the first two outbreaks of Ebola virus (EBOV) occurred. Similarly to Marburg virus, EBOV caused two simultaneous, yet unrelated, outbreaks [2,3]. Even though the filovirus field has progressed tremendously in the last 50 years, there are still no licensed vaccines or treatments approved for human use. However, the situation is rapidly changing as a result of the current outbreak in West Africa. For the first time in the history of filovirus outbreaks, experimental treatments developed in laboratories have been used to treat Ebola-infected health care workers. The first therapeutic used in the 2014–2015 West African outbreak was ZMapp, a cocktail of three monoclonal antibodies developed at the Public Health Agency of Canada in collaboration with Defyrus, the United States Army Medical Research Institute of Infectious Diseases (USAMRIID), Kentucky BioProcessing, and Mapp Biopharmaceutical. ZMapp has been shown to be the most effective postexposure intervention to date in the nonhuman primate (NHP) model, with 100% protection up to five days after the challenge with EBOV [4]. Other therapeutic options that have been used on the field include MIL-77, an antibody-based cocktail closely related to ZMapp, and Favipiravir, a small molecule that interferes with the RNA-dependent RNA polymerase of a variety of viruses including EBOV, convalescent whole blood, and convalescent plasma [5]. Clinical trials for two vaccine candidates, the chimpanzee-adenovirus ChAd3-Zaire Ebola virus (ChAd3-ZEBOV) vaccine and the recombinant vesicular stomatitis virus-Zaire Ebola virus (rVSV-ZEBOV) vaccine, have also been accelerated with the support of the World Health Organization. It is interesting to note that these therapeutics and vaccines are among the few that made it so quickly from the laboratory to the field. WHO and its international partners have invested a considerable amount of effort to fast-track clinical trials for these two vaccines. These efforts were rewarded six months after the outbreak was officially declared, when Phase I clinical trials for the ChAd3-ZEBOV vaccine, developed in the United States, began in the United Kingdom and the US (September 2014), as well as in Mali and Switzerland (October 2014). The rVSV-ZEBOV vaccine, developed in Canada, also began trials in the US (October 2014) and Gabon, Germany, and Switzerland (November 2014), as well as Kenya and Canada (December 2014). Both the ChAd3-ZEBOV and the rVSV-ZEBOV vaccines began Phase III clinical trials at the beginning of 2015. Preliminary results from an open-label, cluster-randomised ring vaccination trial with rVSV-ZEBOV conducted in Guinea showed a vaccine efficacy of 100% [6]. While this number still requires further confirmation, it surely is encouraging. One of the key underlying problems with these therapies and vaccines is that they have been designed to treat or protect against only one virus of the *Filoviridae* family, which is currently composed of eight distinct viruses and many more variants. This review will focus on the evolution of filoviruses, with an in-depth look into the *Ebolavirus* genus and the impact that this evolution may have on current therapies and vaccines in development.

## Ebolavirus

**Fig 1:**
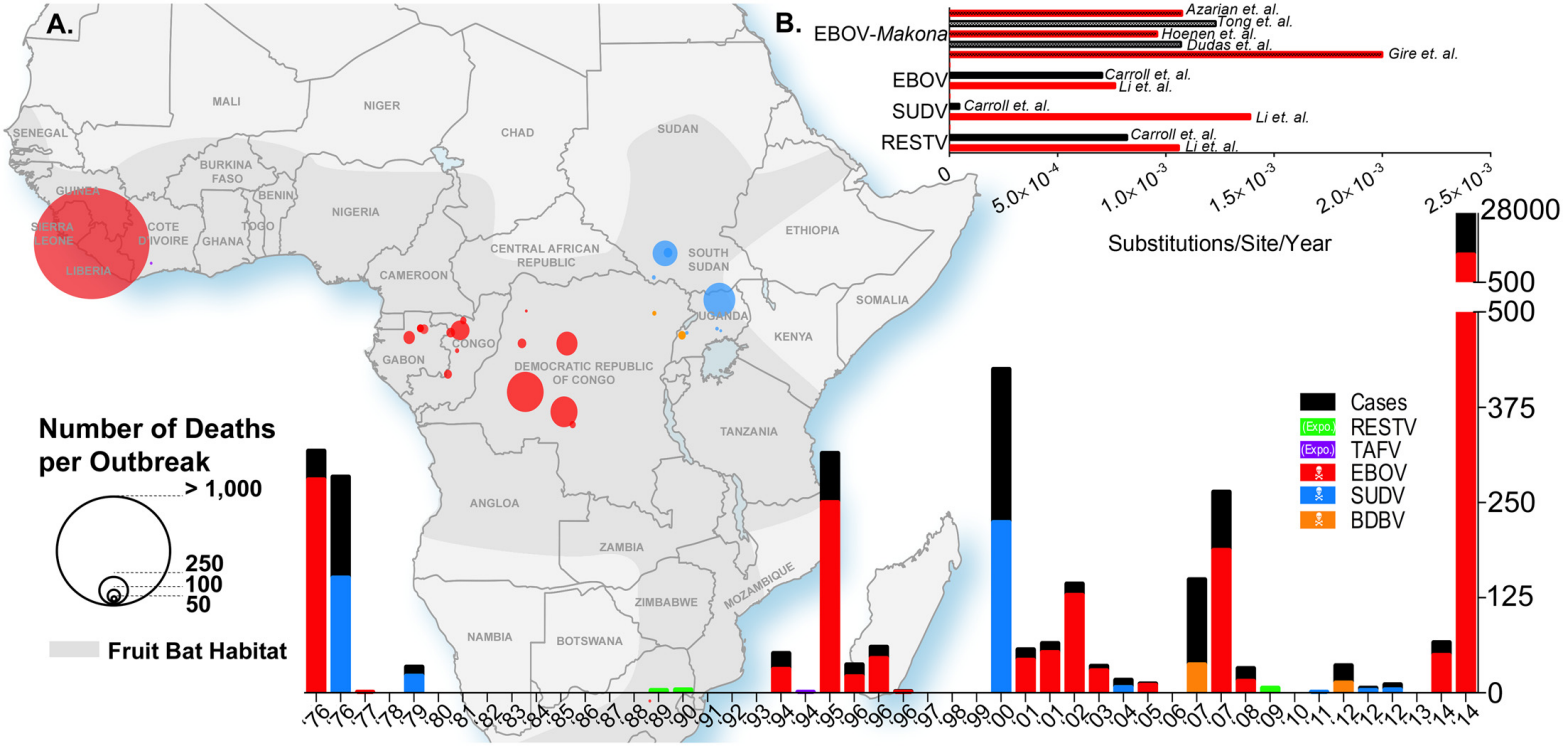
Ebolavirus outbreaks past and present. (A) The geographic map of Africa and the bottom histogram illustrate the number of cases, deaths, and the geographic distribution of several Ebola viruses including Reston (RESTV), Tai Forest (TAFV), Ebola (EBOV, formerly Zaire), Sudan (SUDV), and Bundibugyo (BDBV). The histogram in the top right (B) is a review of the calculated evolutionary rates available for EBOV, EBOV-*Makona*, SUDV, and RESTV from various publications.

**Fig 2:**
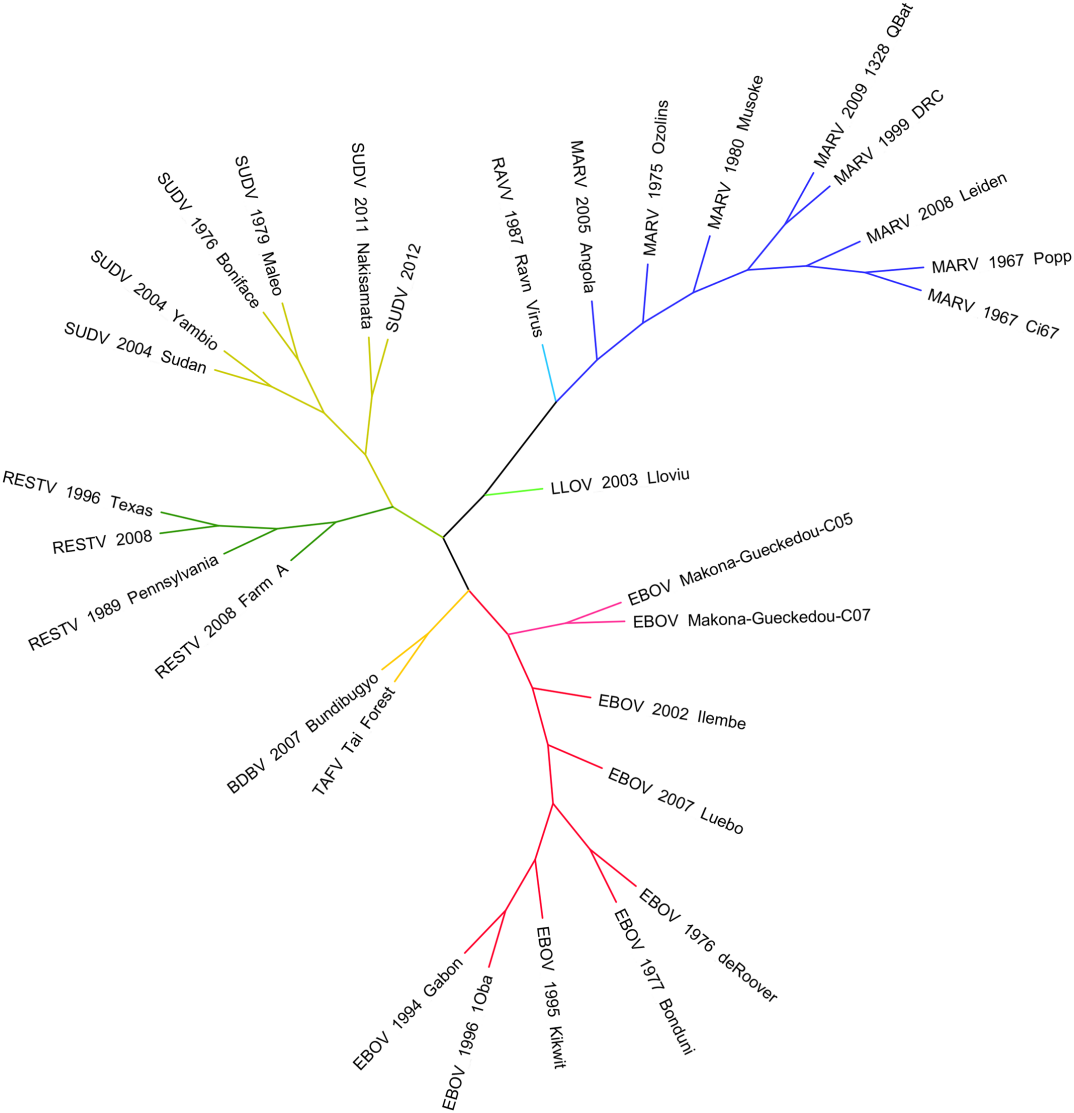
Phylogenetic comparison of *Filoviridae* variants past and present. A phylogenetic analysis was undertaken of *Filoviridae* members from various historical outbreaks as well as the recent 2014–2015 West African outbreak. Thirty-one whole genome sequences were aligned using Clustal Omega 1.2 and visualized in Figtree 1.4.

*Ebolavirus* was the second genus of the *Filoviridae* family to be discovered. It was first identified in 1976 following an outbreak in Zaïre (now known as the Democratic Republic of Congo, or DRC), located in Central Africa, resulting in 318 infections and 280 deaths with a case fatality rate (CFR) of 88% (Fig 1A) [3]. The outbreak was caused by what is now known to be the most lethal species of *Ebolavirus*, *Zaire ebolavirus* (EBOV). This species has caused 15 subsequent outbreaks (including the current West African outbreak and simultaneous outbreak in Boende, DRC) with an average CFR of 79% [7–14]. Simultaneously, an unrelated outbreak occurred in Sudan, located in the northeastern region of Africa, where 284 cases and 151 deaths were reported with a CFR of 53% [2]. This new species, *Sudan ebolavirus* (SUDV), ended up causing six additional outbreaks with an average CFR of 63% [15–19]. In 1994, a new Ebola species, *Taï Forest ebolavirus* (TAFV), was identified in West Africa [20], a considerable geographic distance from previous outbreaks. A scientist practicing an autopsy on a chimpanzee contracted the virus, most likely through handling infected fluids or organs from the infected animal. After receiving treatment in Switzerland, the patient fully recovered and is the only known case of TAFV disease in humans to date. In 2007, 13 years after the identification of the third species of *Ebolavirus*, Uganda became afflicted with another episode of viral hemorrhagic fever. There were 37 reported deaths over a total of 149 cases, for a CFR of 25%. This time, the causative agent was a genetically distinct species of *Ebolavirus*, *Bundibugyo ebolavirus* (BDBV) [21], the least lethal species of *Ebolavirus* in humans as of now, with an average CFR of 38% from documented outbreaks. There has been only one other outbreak caused by this species, which was in 2012 in the DRC [15]. The last species of the genus, *Reston ebolavirus* (RESTV), lies in a different category from the four species previously mentioned, due to its lack of pathogenicity in humans to date. This species has surfaced on seven different occasions, infecting NHPs, pigs, and humans [22–29]. RESTV is capable of inducing disease in NHPs, and more recently was shown to cause asymptomatic infection in pigs [30,31]. The 2014–2015 West African outbreak was caused by the EBOV variant Makona (Fig 2). This variant was found to belong to a different clade of EBOV; however, this clade is in a sister relationship with the other known EBOV sequences [32]. This would suggest a parallel evolution from a common ancestor, with variants from the DRC or Gabon. As to why fatality rates vary between species, that mystery remains at least partially unsolved to this day. Various outbreak settings and levels of clinical care over time make the analysis difficult. From an evolutionary point of view, the various levels of pathogenicity could be explained by various mutations in any of EBOV’s seven genes. Not enough is actually known about the precise mechanism of action of each protein of the virus and how variation in those proteins can attenuate or enhance pathogenicity, compared to other viruses such as influenza or the human immunodeficiency virus (HIV). However, many studies have linked different genes to functions associated with virulence [33–40]. For example, the glycoprotein (GP) gene has been linked to cytotoxicity and virulence, although not sufficiently to explain some of the important dichotomies between contrasting species such as Zaire and Reston [41–44]. Similarly, VP24 and VP35 are well-known inhibitors of innate immune signalling pathways, but the extent of the molecular disparities between the different species and variants has never been assessed directly [45]. The amount of clinical data collected during this outbreak may now provide some understanding as to the exact determinants of fatality rates and pathogenicity between species.

### Evolutionary rate

The process through which various species of *Ebolavirus* have evolved remains unclear to this day. However, recent advances in next-generation sequencing (NGS) will allow for the unprecedented evolutionary analysis needed to understand *Ebolavirus* evolution. Early sequencing in the mid-1990s allowed for the establishment of the first phylogenetic tree to shed light on the relationship between different variants of the first four *Ebolavirus* species that had been discovered at that time (*Bundibugyo ebolavirus* had not been identified yet) as well as two viruses of the *Marburg marburgvirus* species [46]. This tree was based on the sequencing of the GP gene, which is the least conserved between *Filoviruses* [47]. Some reports suggest that the polymerase (L) gene should be used as a reference gene, as it is often thought to be the most conserved gene within most viruses. However, Sanchez et al. showed in 2005 that this was not the case for *Ebolaviruses*, and that VP40 and VP24 were conserved to an even greater extent between species than the L gene [47]. As mentioned by Li et al., it is unclear, however, if using GP as a reference sequence is the best option [48]. It was shown previously that the difference between the evolutionary rates of GP and L for EBOV was not statistically significant, with ~8.0 x 10^−4^ versus 6.2 x 10^−4^ substitutions per site per year, respectively [49,50]. In 2014, Li et al. established that the evolutionary rates for the same two genes were 13.94 x 10^−4^ and 23.14 x 10^−4^ substitutions per site per year for EBOV [48], a difference which was also not statistically significant. These studies establish that either gene could be suitable for phylogeny and evolutionary analysis of EBOV, even though GP is less conserved between species and variants and mutates at a similar rate as L. Based on that conclusion, it was estimated by Li et al. that the evolutionary rates were 7.66 x 10^−4^, 13.94 x 10^−4^, and 10.61 x 10^−4^ substitutions/site/year for EBOV, SUDV, and RESTV, respectively (Fig 1B) [48]. These rates are in keeping with those observed by Carroll et al. with the exception of SUDV (7.06 x 10^−4^, 0.46 x 10^−4^, and 8.21 x 10^−4^ substitutions/site/year for EBOV, SUDV, and RESTV, respectively) [51]. Some of the minor disparities in these studies could be explained by the use of only GP sequences by Li et al., whereas Carroll et al. based their findings on full genome sequences. One explanation for the drastic differences observed in evolutionary rates reported for SUDV could be the number of sequences included in the analyses. Carroll et al. included the sequence from the 2011 SUDV outbreak in Uganda, whereas Li et al. only included sequences up to the 2004 outbreak in Sudan. Of note, TAFV and BDBV were excluded from both analyses because of the lack of available sequences. These results are also in accordance with the analysis conducted by Walsh et al. that established the evolutionary rate of EBOV to be 9.50 x 10^−4^ substitutions/site/year [49].

Previous work conducted retrospectively after the 1995 outbreak in Kikwit, DRC, revealed that EBOV was genetically stable throughout the outbreak [52], based on the sequencing of a 249-nucleotide region in GP, suggesting that EBOV had been relatively constant in previous outbreak scenarios. Both the 1995 outbreak and the current 2014–2015 outbreak were caused by a single introduction of the virus into the community; however, the 1995 outbreak lasted only six months, which is in stark contrast to the current outbreak [53]. Initial studies established that the 2014–2015 outbreak strain (Makona) had an evolutionary rate between 1.07 x 10^−3^ [54] and ~2.0 x 10^−3^ substitutions/site/year [55]. The latter study by Gire et al. quite alarmingly revealed that the evolutionary rate for the Makona strain could be significantly higher than previous outbreaks. However, a recent study by Hoenen et al. established that the evolutionary rate for Makona, based on full-length genome sequencing of samples from the outbreak in Mali and the available sequences used by Gire et al., was 9.6 x 10^−4^ substitutions/site/year, a value that agrees with previously reported rates [56]. Interestingly, Hoenen et al. found that using the same dataset and analysis parameters used by Gire et al. yielded a rate of 6.9 x 10^−4^, while reanalysis of the sequences published by Gire et al. with a strict molecular clock model yielded a rate of 8.2 x 10^−4^ substitutions/site/year. This highlights the challenging nature of various predictive models for viral divergence and the fact that interpretations from these observations should be taken with caution. Molecular clock models are used to estimate the length of phylogeny divergence. In a strict molecular clock model, it is assumed that the rate of evolution for each branch of the phylogenetic tree is the same, while in a relaxed molecular clock model, that rate can vary among different sections of the phylogeny [57]. The most recent genetics analyses conducted by Tong et al. and Azarian et al. reported evolutionary rates of 1.23 x 10^−3^ (based on whole genome sequences) and 1.075 x 10^−3^ (based on GP sequences) substitutions/site/year, respectively [58,59]. Overall, these results indicate that the Makona variant is not evolving at a higher rate than previous outbreak variants, contrary to initial claims from Gire and colleagues.

### Ancestor and selective pressure

The evolution of *Ebolavirus*, like any other virus, is complex, with many factors playing a role in the selection or elimination of particular variants. Phylogenetic analysis of the most extensively studied species of *Ebolavirus*, *Zaire ebolavirus*, reveals the complexity behind the evolution of this virus. It has been hypothesized for a while now that fruit bats could be the reservoir for *Ebolavirus* [60–62]. This hypothesis was considered because some specimens were found to be polymerase chain reaction (PCR)-positive and/or seropositive for antibodies against EBOV. It is important to note, however, that no live, replication-competent EBOV has ever been isolated from bats, unlike Marburg virus [63]. Interestingly, the sequencing of the L gene of bat-derived EBOV showed that it could have recently experienced a genetic bottleneck-type event [50], because the common ancestry of these sequences seems to be in opposition with the notion that EBOV and bats have been interacting together for an extended period of time. Based on the sequencing of EBOV GP, it was previously identified that all viruses causing outbreaks since 2001 had a most recent common ancestor circa 1999. Biek et al. have put forth several alternative scenarios that could explain this genetic bottleneck [50]. The first scenario that the authors raised to explain this bottleneck is that the bat population at this time decreased significantly, causing a reduction in the viral population size. Alternatively, infected bats could have introduced EBOV in the region near Congo and Gabon circa 1999. Another scenario is that the fruit bat species identified by Leroy et al. is not the primary reservoir, making it possible that EBOV was introduced by other species and emerged in the human population.

The evolutionary history of *Ebolaviruses* would not be complete without discussing the common ancestors of these viruses. In 1997, when there were only four species identified, Suzuki et al. estimated that EBOV and TAFV diverged about 700 to 1,300 years ago, that SUDV and RESTV diverged about 1,400 to 1,600 years ago, and that these two clusters diverged about 1,000 to 2,100 years ago [64]. The same analysis was conducted in 2014, but with the sequences of BDBV included. The results indicated that the common ancestor for these five species was about 1,257 years old [48], which is in accordance with the estimations previously made by Suzuki et al. Concerning the three main *Ebolavirus* species (EBOV, SUDV, and RESTV), Li et al. calculated that the times to most recent common ancestor (TMRCA) were 1,971, 1,969, and 1,970 years, respectively [48]. Again, similar results were obtained by Carroll et al. with the exception of SUDV. They estimated that the TMRCA was 1,960 years for EBOV, 1,173 years for SUDV, and 1,979 years for RESTV [51]. As discussed above, SUDV has a much slower evolutionary rate compared to the other species. The 800-year difference in TMRCA for EBOV and RESTV suggests that SUDV is much older. If we accept the fact that EBOV experienced a recent genetic bottleneck, it may just be that we are able to trace the most recent common ancestor further back in time for this species.

To assess the overall selection pressures EBOV, SUDV, and RESTV GP were under, the ratios (ω) between the number of nonsynonymous (d_N_) and synonymous (d_S_) substitutions per site were calculated for each species. A ratio greater than 1 indicates that an amino acid is under increased selection, whereas a ratio less than 1 indicates decreased selection [65,66]. While the results obtained for EBOV, SUDV, and RESTV were 0.229, 0.328, and 0.329, respectively, a secondary analysis by single likelihood ancestor counting (SLAC), fixed effects likelihood (FEL), internal FEL (IFEL), and random effects likelihood (REL) found several distinct amino acid positions in GP under positive selection. Positive results were recorded if they were identified by two of the four methods mentioned above in parentheses. The results varied from one species to another. For EBOV, the analysis revealed that amino acids 377 and 443 were under positive selection; for SUDV, the only hit was amino acid 503, which was observed by REL. For RESTV, the only positive results were for amino acid 229 [48]. Knowing that GP is involved in receptor binding and membrane fusion [67,68], it was hypothesized that the positive selection of certain amino acids on GP could explain why certain virus lineages gained a broader tropism and were able to infect primates following direct exposure to the virus, possibly through fruit bats [69]. However, considering the reduction of genetic diversity since 1970 in EBOV [48], the viruses that managed to survive in those animal reservoirs could potentially be the last remaining lineages of EBOV. It is possible that only a very short window is available for filoviruses to jump in and replicate within the human population. This window could be the result of extreme pressures, which could suggest an adaptation to a distant host that keeps these viruses unsuitable for a more symbiotic relationship in the human environment.

It is safe to say that because of the ongoing outbreak, the potential for increased evolution of EBOV and positive selection of epitopes (particularly within the EBOV GP) may lead to a reduced efficacy of the current therapies undergoing clinical testing. A recent analysis by Kugelman et al. identified 21 nonsynonymous mutations within GP comparing the EBOV Kikwit variant (used to generate ZMapp) and the EBOV Makona West African outbreak variant [70]. The ZMapp cocktail tolerates 18 of these mutations; however, three new mutations, and an additional one identified by Azarian et al., have evolved since the outbreak began and have yet to be evaluated for resistance [59]. In addition, there has only been one documented in vivo case of escape from ZMAb [71,72], the predecessor of ZMapp. Thus, monitoring the continued evolution of EBOV during the outbreak will be critical for the future efficacy of ZMapp. However, ZMapp targets several GP epitopes, which may cause EBOV to generate excessive GP mutations at the cost of viral fitness, reducing the likelihood of an EBOV variant completely circumventing treatment.

## Concluding Remarks

While the current outbreak seems to be waning, there is always the possibility of resurgence. The apparition of a new epicenter in any of the affected countries could be due to a mutated variant of the original Makona or because of the introduction of an unrelated virus. In both scenarios, rapid identification of the virus as well as full genome sequencing will be vital for understanding what threat the international community is facing. Future outbreaks are also to be considered, opening the discussion of how new Ebola variants may affect current and future vaccines and therapies. Broader protective and curative options will need to be considered as new variants keep emerging. The discovery of a new filovirus in 2011 [73] is a reminder that this family of viruses still has a few tricks up its sleeve, and that targeted therapeutics and vaccines may not be enough in the eventuality that a new virus or variant ever spreads in humans. Alternatively, the current outbreak has not led to the emergence of a significantly divergent virus with obvious new characteristics, despite the extraordinary number of passages in thousands of humans. Taken together, these observations point at a relatively stable evolution of an emerging Ebola virus variant in the human population during the current West African outbreak.
